# Analysis of Nutrients and Volatile Compounds in Cherry Tomatoes Stored at Different Temperatures

**DOI:** 10.3390/foods12010006

**Published:** 2022-12-20

**Authors:** Dan Wang, Yujiao Wang, Zhenzhen Lv, Zhiming Pan, Yunlu Wei, Chang Shu, Qingxiao Zeng, Yinnan Chen, Wen Zhang

**Affiliations:** 1 School of Life Science and Engineering, Southwest University of Science and Technology, Mianyang 621010, China; 2 Deyang Food and Drug Safety Inspection Center, Deyang Administration for Market Regulation, Deyang 618000, China; 3 College of Biosystems Engineering and Food Science, Zhejiang University, Hangzhou 310058, China; 4 Key Laboratory of Intelligent Equipment and Robotics for Agriculture of Zhejiang Province, Hangzhou 310058, China

**Keywords:** cherry tomato, kinetic model, volatile compounds, freshness discrimination

## Abstract

Monitoring of the quality change of cherry tomatoes during storage is very important for the quality control of cherry tomatoes. In this study, the soluble solids content (SSC), reducing sugars (RSs), titratable acids (TAs), ascorbic acid (AA) and lycopene of cherry tomatoes during storage at 0, 4, 10 or 25 °C were measured, and the kinetic models were established. The results showed that the zero-order reaction combined with the Arrhenius kinetic model could be used for the prediction of changes in SS, RS and AA content. The first-order reaction combined with the Arrhenius kinetic model could be used for the prediction of changes in the TA and lycopene content. The volatile compounds of cherry tomatoes were simultaneously determined by the gas chromatography–mass spectrometry (GC–MS) and electronic nose (E-nose). A total of 104 volatile compounds were identified by GC–MS. Orthogonal partial least squares discriminant analysis (OPLS-DA) showed that there were 13 different metabolites among cherry tomatoes with different freshness. The accuracies of Fisher’s models based on E-nose for discriminating freshness of cherry tomatoes stored at 0, 4, 10 and 25 °C were 96%, 100%, 92% and 90%, respectively. This study provides a theoretical basis for the quality control of cherry tomatoes during storage.

## 1. Introduction

Cherry tomatoes (*Solanum lycopersicum* var. *cerasiforme*), known as sainfoin and pearl tomatoes, belong to the tomato genus Solanaceae and are one of the most popular varieties of tomatoes [1]. Cherry tomatoes are red in the fresh state, round in shape, and have a special aroma [2]. Compared with ordinary tomatoes, cherry tomatoes are richer in organic acids, ascorbic acid (AA), lycopene, beta-carotene and other nutrients, e.g., the AA content of cherry tomatoes is 1.7 times higher than that of ordinary tomatoes [3,4,5]. The cherry tomato is a kind fruit with certain health benefits, such as improving immunity, slowing down aging, lowering blood pressure, lowering cholesterol and preventing cancer [6,7]. The cherry tomato is a kind of indispensable fruit in people’s lives for its high nutritional and economic value.

However, cherry tomatoes are respiratory climacteric fruit and are susceptible to rot during storage due to their rapid ripening or pathogenic invasion, which makes it more difficult to control their quality during storage [8]. Lycopene and AA, as the key nutrients of cherry tomato, are easily decomposed by temperature [9,10]. Therefore, the storage temperature is an important factor to maintain the nutritional quality of cherry tomatoes. However, few studies have focused on the nutritional quality of cherry tomatoes under different storage temperatures. The Arrhenius equation has been widely applied to quantify the effect of temperature on the rate of several chemical and biochemical reactions [11,12]. Accurate knowledge of the kinetic parameter is essential to predict the change in the nutritional quality of cherry tomatoes during storage.

Aroma is one of the most important characteristics of fruit. Moreover, aroma is a complex mixture of volatile compounds that are critical to the flavor of cherry tomatoes and, along with sugar and acid, play a key role in consumer acceptance of tomatoes [13]. Fruit present different volatile compounds at different stages of storage. The appearance of some volatiles has an impact on the aroma quality of cherry tomatoes, thus affecting the acceptability. The identification of volatile compounds in cherry tomatoes during storage is of great significance for the classification of the freshness grade. Few studies have focused on the identification of the freshness of cherry tomatoes by volatile compounds. Moreover, the difference of volatile compounds in cherry tomatoes at different storage stages is not clear.

Electronic nose (E-nose) and gas chromatography-mass spectrometry (GC–MS) are widely used for the analysis of volatile compounds of fruit and vegetables [14,15]. E-nose can provide rapid sensory information for food shelf-life prediction, freshness assessment and monitoring, but E-nose cannot provide the qualitative and quantitative analysis of volatile compounds [16,17]. GC–MS is widely used for the qualitative and quantitative analysis of volatile components. However, the complicated pretreatment is usually required for GC–MS. The long pretreatment time makes it so that it cannot be used for the rapid detection [18]. The combined application of E-nose and GC–MS allows for a better evaluation of the freshness of cherry tomatoes. Therefore, E-nose combined with GC–MS was used to determine the volatile compounds of cherry tomatoes and identify the freshness of cherry tomatoes in this study.

The objectives of this study were to (1) investigate the changes in nutrients and volatile compounds of cherry tomatoes stored at different temperatures; (2) investigate the feasibility of kinetic models for describing the nutrients change of cherry tomatoes; (3) screen the differential metabolites for distinguishing different freshness of cherry tomatoes by GC–MS; and (4) identify the flavor differences of cherry tomatoes with different freshness by E-nose and establish discrimination models for the freshness of cherry tomatoes. 

## 2. Materials and Methods

### 2.1. Samples

Cherry tomatoes [*Lycopersicon Esculentum* var. *Cerasiforme* Alef] were cultivated by using standard agricultural practices at Yuquanwa Farm in Weifang, Shangdong province, China. Cherry tomatoes were harvested by hand about 40 d after fructifying from 26 to 27 October 2021. Samples without mechanical damage, insect pets or plant diseases were selected, and then were transported to the laboratory. After storage at room temperature for 24 h, some samples were selected for immediate determination, and the rest were immediately stored at 0, 4, 10, or 25 °C (85–90% RH). The determination of nutrients met 3 biological parallel requirements. The determination of volatile compounds by GC–MS and E-nose met 6 and 10 biological parallel requirements respectively. Except for the samples measured by E-nose, other samples were homogenized up to a puree in a home blender (L10-L191, Joyoung, China) and immediately analyzed. Samples were taken every 3 days at 0 and 4 °C, and every 2 days at 10 and 25 °C for the determination of nutrients and volatile compounds. 

### 2.2. Reagents

The standard product 2-nonanone used in this experiment was provided by the Tanmo Quality Control Standard Material Center.

### 2.3. Measurement of Nutritional Quality 

#### 2.3.1. Soluble Solids Content (SSC)

The homogenate of cherry tomatoes was filtered through 6 layers of gauze and used to measure SSC with a digital refractometer (PR-101a, Atago, Co., Tokyo, Japan). Each treatment was repeated 3 times [19].

#### 2.3.2. Reducing Sugars (RS)

Twenty-five grams of samples were weighed and homogenized, and afterward diluted to 250 mL after adding 5 mL each of zinc acetate solution and potassium ferricyanide solution. The mixture was filtered after standing for 30 min. Five milliliters each of alkaline copper tartrate A and B solutions were absorbed in a 50 mL triangular flask, and afterward 10 mL of water was added. RS content was measured by titration with a sample filtrate. The result was presented on the basis of fresh weight as g kg^−1^.

#### 2.3.3. Titratable Acids (TA)

Fifteen grams of samples were weighed and homogenized. The extraction procedure was similar to that for AA, but distilled water was used instead of the oxalic acid solution. TA, expressed in percentage of citric acid, was measured by the acid–base titration using an automatic titrator (G20, Mettler-Toledo, Zurich, Switzerland), and each treatment was repeated 3 times [20]. The result was presented on the basis of fresh weight as g kg^−1^.

#### 2.3.4. Ascorbic Acid (AA)

Fifteen grams of samples were weighed and homogenized, and then diluted to 100 mL with a concentration of 20 g L^−1^ metaphosphoric acid solution. The mixture was filtered to remove the residues. The AA content was measured by titration with 2,6-dichlorophenolindophenol [21]. The result was presented on the basis of fresh weight as mg kg^−1^.

#### 2.3.5. Lycopene

Lycopene was determined by the visible spectrophotometer method. The standard curve was plotted using Sudan Red I as the standard sample, with 5 replicates for each concentration [22]. The lycopene was quantified by use of a standard linear curve (R^2^ = 0.9994) of lycopene solution in ethyl acetate in concentrations from 0.5 to 2.5 mg L^−1^.Two grams of samples were weighed and homogenized, and then lycopene was extracted with ethyl acetate and the absorbance was measured at 485 nm. The absorbance value was used to calculate lycopene content and each treatment was repeated 3 times. The result was presented on the basis of fresh weight as mg kg^−1^.

### 2.4. Volatile Compounds Detection

#### 2.4.1. GC–MS Conditions

The samples were homogenized separately. Five grams of the samples were taken into a 20 mL glass vial, and then 10 μL of 2-nonanone (0.0113 g L^−1^) was added after adding 1.5 g NaCl. A 50/30 μm DVB/CAR/PDMS (SigmaAldrich, St. Louis, MO, USA) extraction head with sorptive properties for aromatics was selected. According to the instructions for use, the extraction head was required to be aged at 270 °C for 1 h before the initial use, and then activated at 270 °C for 30 min before each use and set aside [23].

Chromatographic separation was carried out on a HP-INNOWax column coated with 100% polyethylene glycol of 60 m × 0.25 mm i.d., 0.25 µm film thickness (Agilent, Inc). The oven temperature started at 40 °C, ramped to 130 °C at a rate of 5 °C min^−1^, ramped to 150 °C at a rate of 3 °C min^−1^, ramped to 230 °C at a rate of 6 °C min^−1^ and held for 2 min. Finally, the oven temperature ramped to 250 °C at a rate of 10 °C min^−1^ with a total run time of 45 min.

Electron impact (EI) mass data from m/z 30 to 500 were acquired and with an ionization voltage of 70 eV. The ion source temperature was maintained at 230 °C. In addition, the temperature of the quadrupole was maintained at 150 °C. The carrier gas was 99.999% high-purity helium, and the flow rate was 1.1 mL min^−1^. 

#### 2.4.2. E-nose Conditions

Individual samples were placed in a 50 mL beaker, sealed with plastic wrap, and allowed standing for 30 min before starting the assay with the E-nose (PEN3, Airsense Analytics GmbH, Schwerin, Germany). 

The E-nose sensor cleaning time was 100 s, and the zeroing time was 5 s. The sample preparation time was 5 s, and the analysis time was 100 s [24]. The flow rate of internal was 250 mL min^−1^, and the flow rate of injection was 250 mL min^−1^.

Freshness was classified according to quality classes as fresh fruit (0, 4, 10 and 25 °C: 0–7, 7, 9 and 9 d), second-fresh fruit (0, 4, 10 and 25 °C: 7–25, 7–22, 9–18 and 9–15 d) and corrupt fruit (0, 4, 10 and 25 °C: 25–31, 22–31, 18–27 and 15–24 d). Fresh fruit and second-fresh fruit were also collectively referred to as edible fruit, which was consistent with the GC–MS partial grouping. The test data with values of 76–78 s were selected for the subsequent analytical process.

### 2.5. Statistical Analysis

#### 2.5.1. Dynamics Modeling

Zero-order and first-order kinetic analyses were performed on the SSC, RS, TA, AA and lycopene content of cherry tomatoes. Zero-order and first-order kinetic models were applied to describe the quality changes during storage of cherry tomatoes with the following equations [25]:

*Q_t_* = *Q*_0_ − *kt* (1)

*Q_t_* = *Q*_0_exp(−*kt*) (2)

where *Q_t_* is the value of nutritional indicators of cherry tomato after storage, *Q*_0_ is the initial value of nutritional indicators, *k* is the reaction rate constant, and *t* is the storage time. Taking the logarithm of Equation (2) to obtain Equation (3),

ln*Q_t_* = ln*Q*_0_ − *kt* (3)

The linear or nonlinear regression equations of nutrient content with storage time were established, and the determination coefficient R^2^ and root mean square error (RMSE) of the equations were obtained. The magnitude of R^2^ and RMSE for each of nutritional indicators at the four temperatures was compared to determining the number of response orders. 

The relationship between the rate of quality deterioration and temperature is usually described by the Arrhenius equation. The Arrhenius equation (Equation (4)) for this reaction was obtained by determining the reaction activation energy (*E_a_*) and calculating the reaction constants.

(4) $$K=A\exp\left(-\frac{E_{a}}{RT}\right)$$

where *A* is the refers to the prefactor, *R* is the universal gas constant about 8.314 J mol^−1^ K^−1^, *E_a_* is the activation energy of the chemical reaction at the test temperature, kJ mol^−1^, and *T* is the absolute temperature. Taking the logarithm of Equation (4) to obtain Equation (5),

(5) $$\ln K=\ln A-\frac{E_{a}}{RT}$$

The Arrhenius equation can evaluate and predict the storage quality change of cherry tomatoes. The storage quality change with time–temperature was quantified so that the quality of cherry tomatoes could be maintained in the range of market acceptance. Equation (6) is the Arrhenius equation corresponding to the zero-order reaction to the quality change of cherry tomatoes during storage:

(6) $$t=\frac{Q_{t}-Q_{0}}{A\exp\left(\frac{-Ea}{RT}\right)}$$

Equation (7) is the Arrhenius equation corresponding to the first-order reaction to the quality change of cherry tomatoes during storage:

(7) $$t=\frac{\ln Q_{t}-\ln Q_{0}}{A\exp\left(\frac{-Ea}{RT}\right)}$$

The models were established using Origin 2021 (OriginLab Corporation, Northampton, MA, USA). The performance of the fitting models was evaluated by the coefficient of determination (R^2^).

#### 2.5.2. GC–MS Data Processing

Compounds were identified by a computer search of NIST Library of spectra carried out by GC–MS, combined with analysis of spectra of compounds reported in relevant literature. Only the results of searches with the similarity index (SI) greater than 80% (maximum 100%) were analyzed in this study. The 2-nonanone was used as the internal standard for the semi-quantitative calculation of the content of each target substance. The content was calculated by Equation (8). The result was presented on the basis of fresh weight as μg kg^−1^:

(8) $$Cx=\frac{1000\times C_{0}\times S_{X}}{10\times S_{0}}$$

where *C_x_* is the unknown volatile compound content/(μg kg^−1^), *C*_0_ is the standard compound content/(μg kg^−1^), *S_x_* is the peak area of the unknown volatile compound/(AU min) and *S*_0_ is the peak area of the added standard compound/(AU min).

The volatile compounds of cherry tomatoes were analyzed using the principal component analysis (PCA), partial least squares-discriminant analysis (PLS-DA) and orthogonal partial least squares discriminant analysis (OPLS-DA) models using SIMCA-P 13.0 software. The PLS-DA model was validated using the permutation test (*n* = 200 replicates). The OPLS-DA model was validated using the variance analysis of the cross-validated residuals (CV-ANOVA), and the model was validated with *p* < 0.05, indicating that the model was valid. In addition, the *t*-test and the correlation analysis were performed using SPSS 25.0 software (SPSS, Inc., Chicago, IL, USA) and Origin 2021 software (OriginLab Corporation, Northampton, MA, USA). Substances that met the conditions of the *t*-test (*p* < 0.05), PLS-DA and OPLS-DA models (VIP > 1) were considered the potential differential metabolites.

#### 2.5.3. E-nose Data Processing

The E-nose data results were analyzed using Origin 2021 software for PCA and LOA. Fisher’s discriminant model was then developed using SPSS 25.0 software.

## 3. Results and Discussion

### 3.1. Effect of Storage Temperature on Nutritional Quality of Cherry Tomatoes

**Figure 1:**
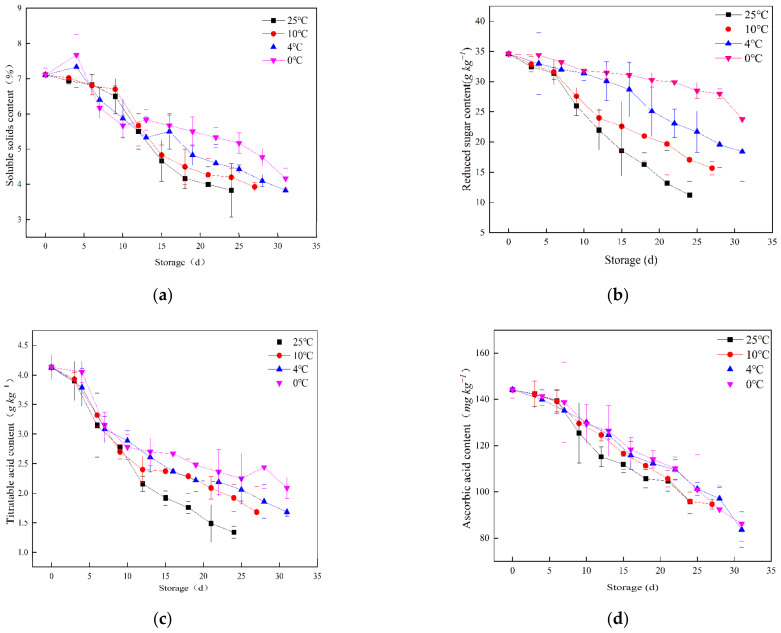

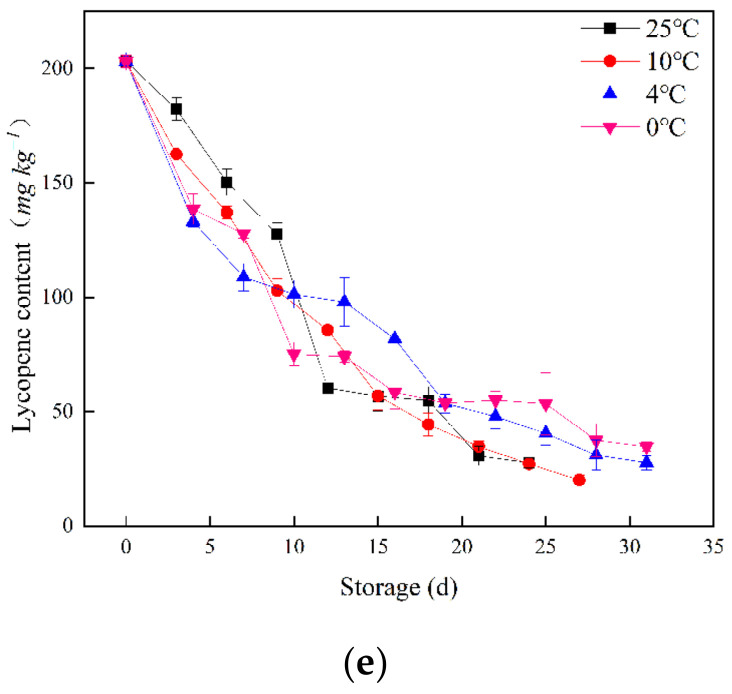
Changes in soluble solids content (**a**), reducing sugars (**b**), titratable acids (**c**), ascorbic acid (**d**) and lycopene (**e**) of cherry tomatoes during storage.

Figure 1 shows the changes in SSC, RS, TA, AA and lycopene content. All nutritional indicators showed a decreasing trend during storage at different storage temperatures. The rate of decline was faster when the temperature was higher. For example, the decreasing rate of each nutritional indicator was higher at 10 and 25 °C, while the decreasing rate was lower at 0 and 4 °C. 

SSC, RS and TA are important indices for evaluating the taste and flavor of cherry tomatoes. SSC, RS and TA all showed a downward trend. SSC decreased from approximately 7% to 4% while RS decreased from approximately 35 to 15 g kg^−1^ during storage (Figure 1a,b). TA decreased from approximately 4 to 1.5 g kg^−1^ during storage (Figure 1c). Since cherry tomatoes were fully ripe when they were picked, most of the starch had been converted to RS. With the extension of the storage time, the RSs in the fruit were consumed by the respiration of the fruit, while SSC and RS decreased simultaneously. At the same time, organic acids as direct oxidation substrates were continuously decomposed, resulting in a decrease in TA content [26].

Cherry tomatoes are nutrient-rich for their high level of AA and lycopene. However, AA and lycopene are extremely unstable and decline fast at higher temperatures. AA decreased from approximately 140 to 85 mg kg^−1^, while lycopene decreased from approximately 200 to 30 mg kg^−1^ during storage (Figure 1d,e). This is consistent with the previous research results that the increase in storage temperature can significantly increase the degradation of lycopene and AA [10,27,28]. The degradation of lycopene is mainly affected by light and heat treatment [29,30]. The degradation of AA is mainly caused by physical dissolution and oxidative decomposition [31].

The results showed that the changes in nutritional quality of cherry tomatoes were temperature-dependent and the degradation rate of each nutrient component increased with the increase in storage temperature. Therefore, low temperature is an effective method to limit the nutrient loss of cherry tomatoes.

### 3.2. Effect of Storage Temperature on Volatile Compounds of Cherry Tomatoes

**Figure 2:**
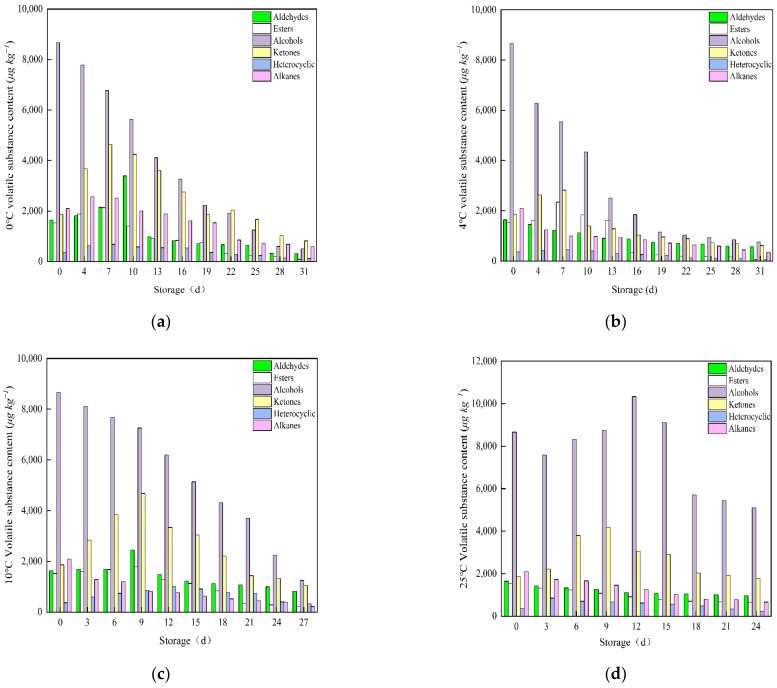
Changes in volatile compounds contents of cherry tomatoes stored at 0 (**a**), 4 (**b**), 10 (**c**) or 25 °C (**d**).

Figure 2 shows the changes in the relative contents of volatile component in cherry tomatoes stored at 0, 4, 10, or 25 °C. Table S1 shows 104 volatile compounds identified during the storage of cherry tomatoes. The content of volatile compounds gradually decreased with the increase in storage time. The main body of volatile compounds was different in different periods at different storage temperatures. The volatile compounds of cherry tomatoes are mainly alcohols, aldehydes, esters and ketones during storage, which was consistent with the results of Selli et al. [32]. The contents of alcohols, aldehydes and esters decreased with the increase in storage time. Alcohol decreased from approximately 8663.75 to 1901.57 μg kg^−1^ while aldehydes decreased from approximately 1636.06 to 662.58 μg kg^−1^ during storage. Esters decreased from approximately 1523.28 to 507.30 μg kg^−1^ during storage. The content of ketones first increased and then decreased during storage. The ketones reached the maximum value on day 7 at 0 and 4 °C, and on the day 9 at 10 and 25 °C. Carotenoids are precursors for the formation of most key ketones in cherry tomatoes [33]. The enzyme activity of cherry tomatoes stored under 25 °C was maintained well at the early stage of storage, promoting the conversion of carotenoids to ketones. Therefore, ketones had a brief upward trend at the beginning of storage.

The results showed that the types and contents of volatile compounds of cherry tomatoes under storage conditions with lower temperatures (0 and 4 °C) were higher than those under storage conditions at room temperature (10 and 25 °C). However, the final contents of major volatile compounds were lower than those under storage conditions at room temperature (25 °C), indicating that low temperature storage inhibits the spoilage of cherry tomatoes while inhibiting the production of volatile compounds of cherry tomatoes, which was consistent with the results of Maul et al. [34]. In addition, the relative contents of heterocyclic and alkanes decreased slightly with storage. There is little difference in the decreasing degree of other temperatures except 0 °C, indicating that the temperature had less influence on the contents of heterocyclic and alkanes during storage. However, compared with other storage conditions, the relative content and type of esters increased less under 0 °C storage conditions, and the relative content and type of aldehydes decreased more. The result showed that low temperature storage inhibited the production of esters and maintained the content and species of aldehydes. This was because low temperature inhibited the fatty acid metabolism of cherry tomatoes after harvesting, thus inhibiting the conversion of aldehydes to esters, which was consistent with the results of Chen et al. [35]. Therefore, low temperatures could slow the spoilage of cherry tomatoes, but also caused a certain degree of volatile compounds loss.

### 3.3. Modeling the Kinetics of Each Nutrient Component of Cherry Tomatoes at Different Storage Temperatures

#### 3.3.1. Reaction Orders and Rate Constants of Each Nutrient

In order to quantify the change of quality pattern of cherry tomatoes at different storage temperatures, a precise and easy-to-implement storage quality change pattern model was obtained. The kinetic parameters of cherry tomato nutritional quality at different storage temperatures are shown in Table 1. The results showed that the rate constant *k* of nutritional indicators of cherry tomatoes increased with the increase in temperature during storage. The changes in SSC, RS and AA contents were fitted better with the zero-order reaction models (with R^2^ values of 0.7816–0.9917 and RMSE values of 0.0040–1.9940). The changes in TA and lycopene contents were fitted better with the first-order reaction models (with R*^2^* values of 0.8759–0.9935 and RMSE values of 0.0040–2.0630). The results confirmed that both the zero-order and first-order reaction models could well-fit the quality change of cherry tomatoes. 

SSC, RS, AA, TA and lycopene decreased during storage, so *k* values of the zero-order and first-order reaction models for SSC, RS, AA, TA and lycopene were positive. The reaction rate was significantly affected by the temperature (*p* < 0.01), indicating that the temperature is a quite important factor for the storage of cherry tomatoes.

#### 3.3.2. Activation Energy of Nutritional Quality Changes in Cherry Tomatoes

The changes in physicochemical properties of fruit and vegetables during storage are very complex. Simple linear models cannot accurately describe the changes in the quality of fruit and vegetables. The Arrhenius equation is frequently used to describe the relationship between the nutrient retention ratio and temperature in fruit [36]. Determination of degradation kinetic parameters associated with the loss of nutritional quality of fruit and vegetables is essential for establishing the Arrhenius model. Kinetic parameters, such as *k, t*_1/2_, *Q*_10_ and *E_a_,* are commonly used to estimate the shelf life of agro-products [25]. Figure S1 shows the lnk−1/T plots of SSC, RS, TA, AA and lycopene content. Table 2 shows *E_a_* and the value that refers to the prefactor (*A*) obtained from the slope and intercept of the straight lines, respectively. *E_a_* reflects the heat required to be absorbed from the external environment for the chemical reactions occurring during the quality deterioration of cherry tomatoes, which is important for temperature selection and control in cherry tomatoes storage. The lower *E_a_* is, the higher the reaction rate will be [37]. Table 2 shows that the reaction rate of AA was largest during the storage of cherry tomatoes, indicating that this indicator is susceptible to change, followed by lycopene, SSC, TA and RS (with *E_a_* values of 4.99 × 10^3^–2.84 × 10^4^ kg mol^−1^). *E_a_* also responded to the sensitivity of quality changes to temperature. The smaller *E_a_* is, the less sensitive the temperature increase is to quality changes. The results inferred that the temperature sensitivity of AA during storage of cherry tomatoes was lower, and the temperature sensitivity of RS was relatively greater, which was similar to the results of Alves et al. [10].

The Arrhenius equation for quality change during storage of cherry tomatoes can be obtained by bringing *E_a_* and *A* into Equation (6) or (7). The storage quality of cherry tomatoes in a certain period can be predicted according to the initial value, storage time and storage temperature by this equation. For example, the storage time of cherry tomatoes can be estimated by introducing the measured AA content in a certain period of time into the Arrhenius equation.

### 3.4. Determination of Volatile Compounds in Cherry Tomatoes by GC–MS

**Figure 3:**
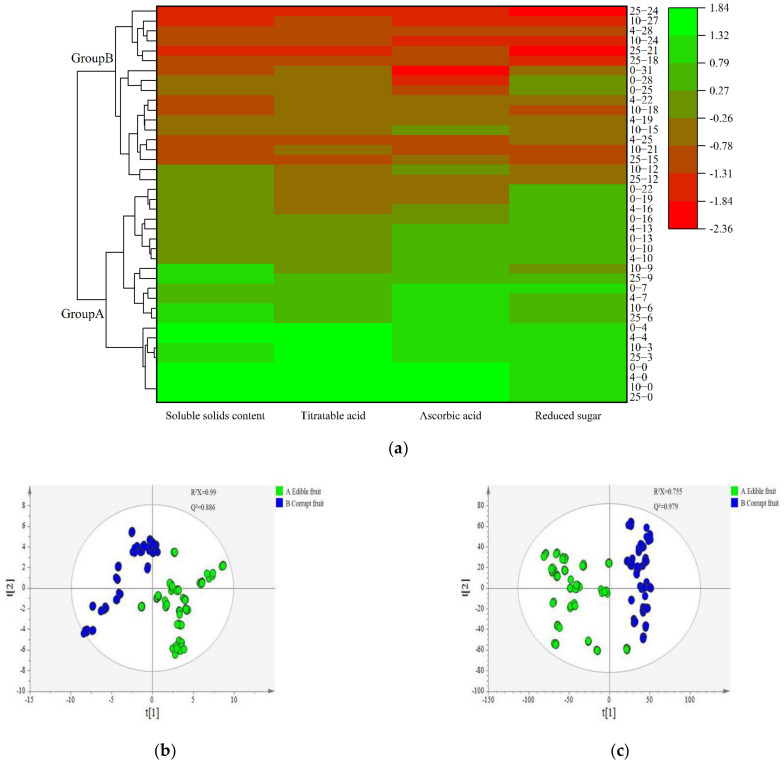

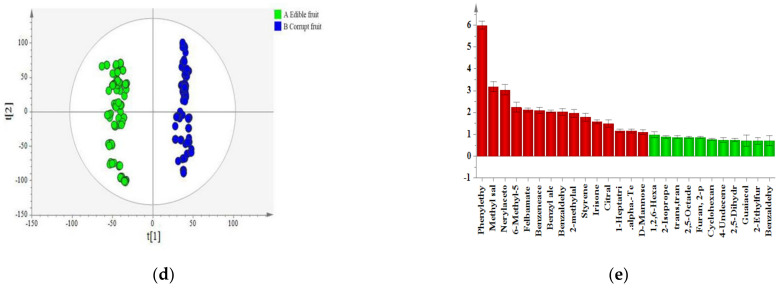
Cluster analysis heat diagram (**a**), PCA score plots based on GC–MS data (**b**), PLS-DA score plots based on GC–MS data (**c**), OPLS-DA score plots based on GC–MS data (**d**) and VIP plots based on GC–MS data (**e**) for cherry tomatoes edible and corrupt fruit. Note: Figure 3a, group A represents the edible fruit and group B represents the corrupt fruit. The first number represents the storage temperature and the number after the horizontal line represents storage time, e.g., 25–3 indicates cherry tomato samples stored at 25 °C for 3 d.

Cluster analysis is widely used to classify substances [38]. Cluster analysis aggregates samples according to the similarity degree of quality characteristics, and a higher similarity is preferred to aggregate. Figure 3a shows the cluster analysis heat diagram of the freshness grades of cherry tomatoes. Cherry tomatoes were grouped using a cluster analysis based on four nutritional indicators, including the SSC, AA, RS, and TA value. Nutrient indices measured at different storage temperatures were divided into two parts by the cluster tree axis. The figure showed that days 22, 16, 9 and 9 were divided into the first group at 0, 4, 10 and 25 °C, respectively, and the remaining storage time was the second group. The results showed that the grade of cherry tomatoes was divided into group A (edible fruit) and group B (corrupt fruit) by the cluster analysis.

The volatile compounds were grouped according to the clustering results. Figure 3b shows the PCA score plots of cherry tomatoes based on GC–MS data. The models were fitted well with R^2^X of 0.99 and Q^2^ of 0.886. The distribution areas of cherry tomatoes at the different degrees of freshness were independent of each other, which indicated that the volatile compounds changed in the storage process. The results showed that to group volatile compounds by the changes in nutrient indices was feasible.

Figure 3c shows the PLS-DA score plots of cherry tomatoes based on GC–MS data. The models were fitted well with R^2^X of 0.755 and Q^2^ of 0.979. The cherry tomatoes of edible and corrupt fruit were in different regions of the ellipse, and the duplicate samples within each group were clustered, indicating that the metabolites of edible and corrupt fruit were significantly different. Therefore, the PLS-DA model could be used to distinguish the cherry tomatoes with different degrees of freshness.

Figure 3d shows the OPLS-DA score plots of cherry tomatoes based on GC–MS data. The cherry tomatoes of edible and corrupt could be clearly separated, indicating that there were significant differences in the volatile compounds between edible and corrupt cherry tomatoes. This was consistent with the result obtained by the PLS-DA model. The classification effect of the OPLS-DA model was better because the OPLS-DA model filters out the noise, which was irrelevant to the classification information. 

The VIP value of the OPLS-DA variable importance projection can quantify the contribution of each variable to the classification. As shown in Figure 3e and Table 3, a total of 15 differential metabolites (VIP > 1) were screened. Then, the *t*-test was performed on the 15 differential metabolites to verify whether they were statistically different between the edible and corrupt fruit. After excluding variables with a *p* value greater than 0.05, 13 potential differential metabolites were finally screened, including phenylethyl alcohol, methyl salicylate, felbamate, benzeneacetaldehyde, benzyl alcohol, benzaldehyde, 2-methylallyl 2-methylbutyrate, styrene, irisone, citral, 1-heptatriacotanol, alpha-terpineol, and d-mannose.

The results showed that the d-mannose, felbamate, benzeneacetaldehyde, benzyl alcohol, 2-methylallyl 2-methylbutyrate, and 1-heptatriacotanol are found only in edible fruits. The contents of phenylethyl alcohol, methyl salicylate, benzaldehyde, styrene and citral were high in edible fruit, but low in corrupt fruit. The contents of irisone and alpha-terpineol were low in edible fruit, but high in corrupt fruit.

**Figure 4:**
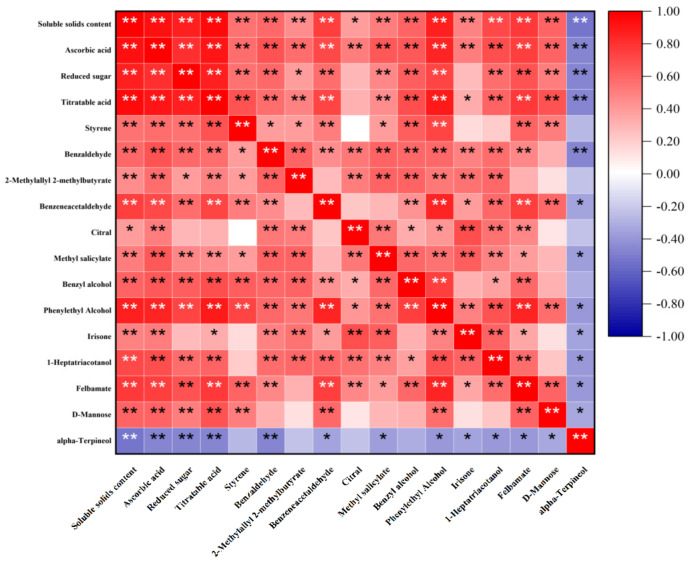
Correlation analysis between the potential differential metabolites and nutritional indicators of cherry tomatoes. Note: “**” indicates a highly significant difference (*p* < 0.01) and “*” indicates a significant difference (*p* < 0.05).

Figure 4 shows the correlations between the 13 potential differential metabolites with SSC, AA, RS, and TA. Alpha-terpineol was negatively correlated with SSC, AA, RS, and TA, and the remaining substances were positively correlated with the four nutritional indicators (*p* < 0.05). Moreover, 2-methylallyl 2-methylbutyrate and citral were significantly and positively correlated with RS (*p* < 0.05), and irisone and citral were significantly and positively correlated with TA (*p* < 0.05). The remaining substances were highly significant positively correlated with the four nutritional indicators (*p* < 0.01). Therefore, the 13 substances were considered as metabolites of differences between the edible and corrupt fruit.

### 3.5. Determination of Volatile Compounds in Cherry Tomatoes by E-nose

#### 3.5.1. PCA Analysis of Freshness of Cherry Tomatoes at Different Storage Temperatures

**Figure 5:**
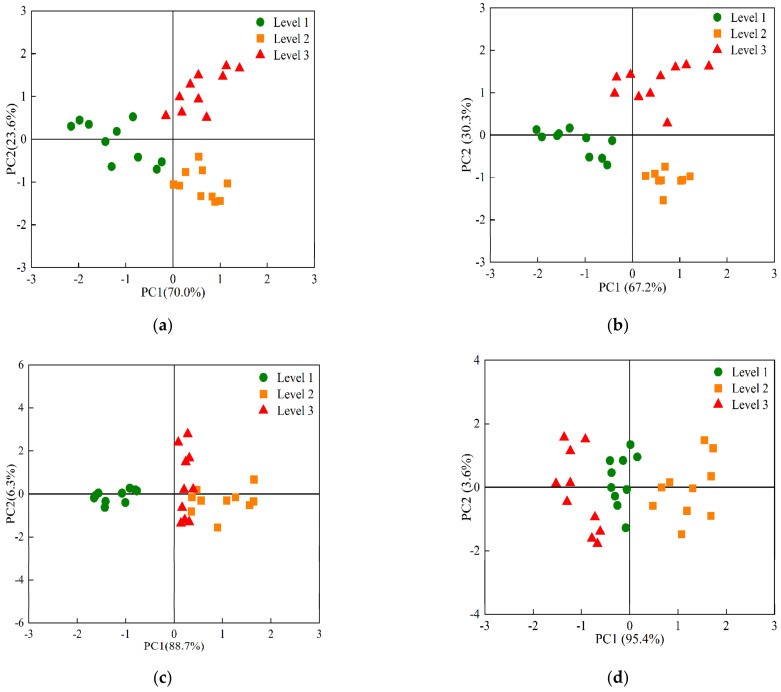
PCA score plots of cherry tomatoes with different freshness based on E-nose data at 0 (**a**), 4 (**b**), 10 (**c**) and 25 °C (**d**). Note: Level 1: fresh fruit, Level 2: second-fresh fruit, Level 3: corrupt fruit.

PCA analysis was conducted to show the difference among samples based on E-nose data. Figure 5 shows the PCA score plots of cherry tomatoes with different freshness based on E-nose data at 0, 4, 10 and 25 °C. The total contributions of the first principal components (PC1) and second principal components (PC2) at 0, 4, 10 and 25 °C were 93.6%, 97.5%, 95% and 99%, respectively. In general, the principal components (PCs) can represent the original data when the accumulated contribution of certain PCs is over 85% [39]. The first two PCs accounted for more than 90% and were used in this study. The cherry tomatoes with different freshness were clearly distinguished and had better aggregation within the groups. The models could well-distinguish the volatile compounds of cherry tomatoes at different storage stages. 

Figure S2 shows the load diagram of cherry tomatoes based on E-nose data at 0, 4, 10 or 25 °C. The load diagram allows the analysis of the importance of the E-nose sensors and their contributions to PCA. The load diagram shows the position of the different sensors. The load diagram corresponds with the position of the samples in the score map. The load diagram showed that the sensors with greater contribution were W1S, W1W and W2S in PC1. The main sensitive compounds were methane, hydrogen sulfide and ethanol. The sensors with greater contribution were W1W, W2W and W5S in PC2. The main sensitive compounds were hydrogen sulfide, organic sulfide and aromatic alkanes. The sensors W1W, W2S and W5S were more sensitive to changes in the volatile compounds of cherry tomatoes during storage, which was similar to the results of Feng et al. [40].

#### 3.5.2. Development of Fisher’s Discriminant Model for Freshness of Cherry Tomatoes

Seventy-five cherry tomatoes (including 23 fresh fruit, 28 s-fresh and 24 corrupt fruit) were randomly selected from all the test samples at 0, 4 and 10 °C as the training set. The remaining 35 cherry tomatoes (including 7 fresh fruit, 12 s-fresh and 16 corrupt fruit) were used as the validation set. Sixty cherry tomatoes (including 27 fresh fruit, 13 s-fresh and 20 corrupt fruit) were randomly selected from all the test samples at 25 °C as the training set. The remaining 30 cherry tomatoes (including 13 fresh fruit, 7 s-fresh and 10 corrupt fruit) were used as the validation set.

Table 4 shows the accuracies of the Fisher’s models for discriminating the freshness of cherry tomatoes stored at 0, 4, 10 and 25 °C. The results showed that the discriminant accuracies of the Fisher’s models for discriminating the fresh cherry tomatoes at 0 and 4 °C were 100%, and 95.7% and 96.3% at 10 and 25 °C, respectively. The discriminant accuracies of the Fisher’s models for discriminating the second-fresh cherry tomatoes at 0, 4 and 10 °C were 96.4%, 100% and 96.6%, respectively. However, the discriminant accuracies of the Fisher’s model for discriminating the second-fresh cherry tomatoes at 25 °C was only 76.9%. The discriminant accuracies of the Fisher’s models for discriminating the corrupt fruit at 0, 4, 10 and 25 °C were 91.7%, 100%, 82.6% and 90%, respectively. The accuracies of the Fisher’s models for discriminating the freshness of cherry tomatoes stored at 0, 4, 10 and 25 °C were 96%, 100%, 92% and 90.0%, respectively. The results showed that the Fisher’s models could be used for the discrimination of the freshness of cherry tomatoes.

## 4. Conclusions

The changes in nutritional quality and volatile compounds of cherry tomatoes at different storage temperatures were investigated in this study. SSC, RS, TA, AA and lycopene showed a decreasing trend during storage and the decreasing rate increased with the increase in temperature. The zero-order reaction combined with the Arrhenius kinetic model could be used for the prediction of changes in SSC, RS and AA content of cherry tomatoes. The first-order reaction combined with the Arrhenius kinetic model could be used to predict the changes in TA and lycopene content of cherry tomatoes. The nutrients and volatile compounds of cherry tomatoes were affected by temperature, especially under higher temperatures of 10 and 25 °C. The respiratory metabolism of cherry tomatoes was vigorous, and decay and rot were accelerated under high temperatures. The metabolism of cherry tomatoes was inhibited under lower temperatures of 0 and 4 °C. Therefore, the low-temperature environmental conditions could extend the shelf life of cherry tomatoes.

A total of 104 volatile compounds of cherry tomatoes were identified by GC–MS. A total of 13 volatile compounds were screened as differential metabolites of cherry tomatoes of different freshness, including phenylethyl alcohol, methyl salicylate, felbamate, benzeneacetaldehyde, benzyl alcohol, benzaldehyde, 2-methylallyl 2-methylbutyrate, styrene, irisone, citral, 1-heptatriacotanol, alpha-terpineol, and d-mannose. The volatile compounds of cherry tomatoes varied significantly at different storage periods. The low temperature storage inhibited the spoilage of cherry tomatoes while inhibiting the production of volatile compounds of cherry tomatoes.

An E-nose-based Fisher’s discriminant model for freshness of cherry tomato was developed. The overall discriminant accuracies of the models for the freshness of cherry tomatoes at 0, 4, 10 and 25 °C were 96%, 100%, 92%, and 90%, respectively. This study provides the theoretical basis for the quality control of cherry tomatoes during storage and lays a foundation for the metabolic pathway analysis of cherry tomatoes.

## Figures and Tables

**Table 1:** Rate constants (*k*), coefficients of determination (R^2^) and root mean square error (RMSE) of the zero-order and first-order kinetic regressions for nutritional quality changes in cherry tomatoes at different storage temperatures.

Quality Indices	T (°C)	Zero-Order			First-Order		
		*k*	R^2^	RMSE	*k*	R^2^	RMSE
Soluble solids content (SSC)	0	0.0835	0.9130	0.1810	0.0146	0.9167	0.2248
	4	0.1081	0.9872	0.0040	0.0197	0.9876	0.2457
	10	0.1274	0.9607	0.5920	0.0223	0.9426	0.5176
	25	0.1617	0.9447	0.1690	0.0273	0.9157	0.0040
Reducing sugars (RS)	0	0.0353	0.9192	0.2093	0.0118	0.8973	0.2190
	4	0.0156	0.9591	0.0342	0.0156	0.9335	0.1118
	10	0.0285	0.9916	0.0678	0.0285	0.9914	0.0951
	25	0.0350	0.9676	0.1382	0.0350	0.9253	0.2800
Titratable acids (TA)	0	0.0676	0.7816	0.4582	0.0259	0.8759	0.2040
	4	0.0562	0.8507	0.7410	0.0282	0.9435	0.4712
	10	0.1050	0.8971	0.4300	0.0382	0.9506	0.1120
	25	0.1073	0.9282	0.2160	0.0494	0.9817	0.0080
Ascorbic acid (AA)	0	0.1768	0.9799	0.0258	0.0141	0.9627	0.0869
	4	0.1841	0.9883	0.1930	0.0149	0.9758	0.4360
	10	0.1965	0.9917	0.0865	0.0168	0.9829	0.1720
	25	0.2129	0.9580	0.1040	0.0175	0.9602	0.1270
Lycopene	0	0.4634	0.8130	1.9940	0.0669	0.9694	0.4370
	4	0.5099	0.8840	1.5140	0.0684	0.9720	1.5890
	10	0.6425	0.9496	1.1380	0.0791	0.9935	0.5260
	25	0.7215	0.8667	0.6150	0.0863	0.9574	2.0630

T: temperature.

**Table 2:** Kinetic model of nutritional quality degradation of cherry tomatoes at different storage temperatures.

Quality Indices	T (°C)	R^2^	*A*	*E_a_*/(kg mol^−1^)	Prediction Model
Soluble solids content (SSC)	0	0.989	232.76	1.78 × 10^4^	$t=\frac{Q_{t}-Q_{0}}{e^{5.45}\exp\left(\frac{-17791.96}{RT}\right)}$
	4				
	10				
	25				
Reducing sugars (RS)	0	0.991	10,938	2.84 × 10^4^	$t=\frac{Q_{t}-Q_{0}}{e^{9.3}\exp\left(\frac{-28433.88}{RT}\right)}$
	4				
	10				
	25				
Titratable acids (TA)	0	0.933	104.58	1.88 × 10^4^	$t=\frac{\ln Q_{t}-\ln Q_{0}}{e^{4.65}\exp\left(\frac{-18789.64}{RT}\right)}$
	4				
	10				
	25				
Ascorbic acid (AA)	0	0.967	1.62	4.99 × 10^3^	$t=\frac{Q_{t}-Q_{0}}{e^{0.48}\exp\left(\frac{-4988.4}{RT}\right)}$
	4				
	10				
	25				
Lycopene	0	0.905	1.77	7.40 × 10^3^	$t=\frac{\ln Q_{t}-\ln Q_{0}}{e^{0.57}\exp\left(\frac{-7399.46}{RT}\right)}$
	4				
	10				
	25				

Note: R^2^, coefficient of determination; T, temperature; *E_a_*, energy of activation; *A*, refers to prefactor; $Q_{t}$, the value of a nutritional indicators of cherry tomato after storage; $Q_{0}$, the initial value of nutritional indicators of cherry tomatoes during storage.

**Table 3:** Results of screening potential differential metabolites of cherry tomatoes with varying freshness.

Serial Number	Identified Compounds	VIP	*p*	Significance	PubChem CID
1	Phenylethyl Alcohol	6.000	0.000	**	6054
2	Methyl salicylate	3.190	0.000	**	4133
3	Felbamate	2.120	0.000	**	3331
4	Benzeneacetaldehyde	2.100	0.000	**	998
5	Benzyl alcohol	2.050	0.000	**	244
6	Benzaldehyde	2.040	0.000	**	240
7	2-Methylallyl 2-methylbutyrate	1.960	0.000	**	3,019,320
8	Styrene	1.790	0.004	**	7501
9	Irisone	1.570	0.000	**	5,282,108
10	Citral	1.500	0.000	**	638,011
11	1-Heptatriacotanol	1.160	0.000	**	537,071
12	alpha-Terpineol	1.160	0.002	**	17,100
13	D-Mannose	1.090	0.000	**	18,950

Note: “**” indicates a highly significant difference (*p* < 0.01).

**Table 4:** Results of Fisher’s discriminant function for freshness of cherry tomatoes.

Temperature (°C)	Group	Category	Actual Number of Predicted Samples/Unit			Total Number/pc	Discriminant Accuracy	Total Forecast Rate
			1	2	3			
0	T	1	23	0	0	23	100%	96%
		2	1	27	0	28	96.4%	
		3	2	0	22	24	91.7%	
	V	1	7	0	0	7	100%	97.1%
		2	1	11	0	12	91.7%	
		3	0	0	0	16	100%	
4	T	1	23	0	0	23	100%	100%
		2	0	28	0	28	100%	
		3	0	0	24	24	100%	
	V	1	7	0	0	7	100%	94.3%
		2	2	10	0	12	83.3%	
		3	0	0	16	16	100%	
10	T	1	22	1	0	23	95.7%	92%
		2	1	28	0	29	96.6%	
		3	1	3	19	23	82.6%	
	V	1	6	1	0	7	85.7%	91.4%
		2	1	12	1	14	85.7%	
		3	0	0	14	14	100%	
25	T	1	26	0	1	27	96.3%	90%
		2	1	10	2	13	76.9%	
		3	0	2	18	20	90.0%	
	V	1	12	1	0	13	92.3%	83.3%
		2	0	5	2	7	71.4%	
		3	0	2	8	10	80.0%	

Note: T, training set; V, validation set; 1, 2, and 3 represent Level 1: fresh fruit, Level 2: second-fresh fruit, and Level 3: corrupt fruit.

## Data Availability

Data is contained within the article and Supplementary Materials.

## Supplementary Materials

The following supporting information can be downloaded at: https://www.mdpi.com/article/10.3390/foods12010006/s1. Table S1: Summary of volatile compounds in cherry tomato during storage; Figure S1: Plot of lnk−1/T of soluble solids content (a), reducing sugars (b), titratable acids (c), ascorbic acid (d) and lycopene (e) of cherry tomatoes at different storage temperatures; Figure S2. Load diagram of cherry tomatoes based on E-nose data at 0 (a), 4 (b), 10 (c) or 25 °C (d).

## References

[1] Zhang L., Wang P., Sun X., Chen F., Lai S., Yang H. (2020). Calcium permeation property and firmness change of cherry tomatoes under ultrasound combined with calcium lactate treatment. Ultrason. Sonochem..

[2] Pobiega K., Przybył J.L., Żubernik J., Gniewosz M. (2020). Correction to: Prolonging the shelf life of cherry tomatoes by pullulan coating with ethanol extract of propolis during refrigerated storage. Food Bioprocess Technol..

[3] Zeng C., Tan P., Liu Z. (2020). Effect of exogenous ARA treatment for improving postharvest quality in cherry tomato (*Solanum lycopersicum* L.) fruits. Sci. Hortic.-Amst..

[4] Du W., Olsen C.W., Avena-Bustillos R.J., McHugh T.H., Levin C.E., Mandrell R., Friedman M. (2009). Antibacterial effects of allspice, garlic, and oregano essential oils in tomato films determined by overlay and vapor-phase methods. J. Food Sci..

[5] Yun J., Fan X., Li X., Jin T.Z., Jia X., Mattheis J.P. (2015). Natural surface coating to inactivate Salmonella enterica serovar Typhimurium and maintain quality of cherry tomatoes. Int. J. Food Microbiol..

[6] Wang S., Chu Z., Jia R., Dan F., Shen X., Li Y., Ding X. (2018). SlMYB12 regulates flavonol synthesis in three different cherry tomato varieties. Sci. Rep. UK.

[7] Flores P., Sánchez E., Fenoll J., Hellín P. (2017). Genotypic variability of carotenoids in traditional tomato cultivars. Food Res. Int..

[8] Tang Q., Zhu F., Cao X., Zheng X., Yu T., Lu L. (2019). Cryptococcus laurentii controls gray mold of cherry tomato fruit via modulation of ethylene-associated immune responses. Food Chem..

[9] Hackett M.M., Lee J.H., Francis D., Schwartz S.J. (2004). Thermal stability and isomerization of lycopene in tomato oleoresins from different varieties. J. Food Sci..

[10] Alves J.A., de Cássia Mirela Resende Nassur R., Pires C.R.F., de Alcântara E.M., Giannoni J.A., de Oliveira Lima L.C. (2010). Kinects of vitamin C degradation of ‘palmer’ mangoes (*Mangifera indica* L.) stored at different temperatures. Ciência e Agrotecnologia.

[11] Pinheiro J., Alegria C., Abreu M., Gonçalves E.M., Silva C.L.M. (2013). Kinetics of changes in the physical quality parameters of fresh tomato fruits (*Solanum lycopersicum,* cv. ‘Zinac’) during storage. J. Food Eng..

[12] Giannakourou M.C., Taoukis P.S. (2003). Kinetic modelling of vitamin C loss in frozen green vegetables under variable storage conditions. Food Chem..

[13] El Hadi M.A.M., Zhang F., Wu F., Zhou C., Tao J. (2013). Advances in fruit aroma volatile research. Molecules.

[14] Guo Y., Chen D., Dong Y., Ju H., Wu C., Lin S. (2018). Characteristic volatiles fingerprints and changes of volatile compounds in fresh and dried Tricholoma matsutake Singer by HS-GC-IMS and HS-SPME-GC–MS. J. Chromatogr. B Anal. Technol. Biomed. Life Sci..

[15] Beghi R., Buratti S., Giovenzana V., Benedetti S., Guidetti R. (2017). Electronic nose and visible-near infrared spectroscopy in fruit and vegetable monitoring. Rev. Anal. Chem..

[16] Chen J., Tao L., Zhang T., Zhang J., Wu T., Luan D., Ni L., Wang X., Zhong J. (2021). Effect of four types of thermal processing methods on the aroma profiles of acidity regulator-treated tilapia muscles using E-nose, HS-SPME-GC-MS, and HS-GC-IMS. LWT-Food Sci. Technol..

[17] Baietto M., Wilson A.D. (2015). Electronic-nose applications for fruit identification, ripeness and quality grading. Sensors-Basel.

[18] Dou T., Shi J., Li Y., Bi F., Gao H., Hu C., Li C., Yang Q., Deng G., Sheng O. (2020). Influence of harvest season on volatile aroma constituents of two banana cultivars by electronic nose and HS-SPME coupled with GC-MS. Sci. Hortic. Amst..

[19] Yang B., Guo W., Li W., Li Q., Liu D., Zhu X. (2018). Portable, visual, and nondestructive detector integrating Vis/NIR spectrometer for sugar content of kiwifruits. J. Food Process Eng..

[20] Zhang W., Luo Z., Wang A., Gu X., Lv Z. (2021). Kinetic models applied to quality change and shelf life prediction of kiwifruits. LWT.

[21] Da S.T., Aguiar-Oliveira E., Mazalli M.R., Kamimura E.S., Maldonado R.R. (2017). Comparison between titrimetric and spectrophotometric methods for quantification of vitamin C. Food Chem..

[22] Marković K., Hruškar M., Vahčić N. (2006). Lycopene content of tomato products and their contribution to the lycopene intake of Croatians. Nutr. Res..

[23] Lee J.H.J., Jayaprakasha G.K., Avila C.A., Crosby K.M., Patil B.S. (2019). Metabolomic studies of volatiles from tomatoes grown in net-house and open-field conditions. Food Chem..

[24] Xing M., Sun K., Liu Q., Pan L., Tu K. (2018). Development of novel electronic nose applied for strawberry freshness detection during storage. Int. J. Food Eng..

[25] Kim A., Kim H., Chun J., Heo H.J., Kerr W.L., Choi S. (2018). Degradation kinetics of phenolic content and antioxidant activity of hardy kiwifruit (*Actinidia arguta*) puree at different storage temperatures. LWT.

[26] Lamikanra O., Chen J.C., Banks D., Hunter P.A. (2000). Biochemical and microbial changes during the storage of minimally processed cantaloupe. J. Agr. Food Chem..

[27] Martínez-Hernández G.B., Boluda-Aguilar M., Taboada-Rodríguez A., Soto-Jover S., Marín-Iniesta F., López-Gómez A. (2016). Processing, packaging, and storage of tomato products: Influence on the lycopene content. Food Eng. Rev..

[28] Burdurlu H.S., Koca N., Karadeniz F. (2006). Degradation of vitamin C in citrus juice concentrates during storage. J. Food Eng..

[29] Lambelet P., Richelle M., Bortlik K., Franceschi F., Giori A.M. (2009). Improving the stability of lycopene Z-isomers in isomerised tomato extracts. Food Chem..

[30] Shi J., Dai Y., Kakuda Y., Mittal G., Xue S.J. (2008). Effect of heating and exposure to light on the stability of lycopene in tomato purée. Food Control..

[31] Robertson G.L., Samaniego C.M.L. (1986). Effect of initial dissolved oxygen levels on the degradation of ascorbic acid and the browning of lemon juice during storage. J. Food Sci..

[32] Selli S., Kelebek H., Ayseli M.T., Tokbas H. (2014). Characterization of the most aroma-active compounds in cherry tomato by application of the aroma extract dilution analysis. Food Chem..

[33] Wang L., Baldwin E.A., Bai J. (2016). Recent advance in aromatic volatile research in tomato fruit: The metabolisms and regulations. Food Bioprocess Technol..

[34] Maul F., Sargent S.A., Sims C.A., Baldwin E.A., Balaban M.O., Huber D.J. (2000). Tomato flavor and aroma quality as affected by storage temperature. J. Food Sci..

[35] Chen G., Hackett R., Walker D., Taylor A., Lin Z., Grierson D. (2004). Identification of a specific isoform of tomato lipoxygenase (TomloxC) involved in the generation of fatty acid-derived flavor compounds. Plant Physiol..

[36] Nisha P., Singhal R.S., Pandit A.B. (2011). Kinetic modelling of colour degradation in tomato puree (*Lycopersicon esculentum* L.). Food Bioprocess Technol..

[37] Remini H., Mertz C., Belbahi A., Achir N., Dornier M., Madani K. (2015). Degradation kinetic modelling of ascorbic acid and colour intensity in pasteurised blood orange juice during storage. Food Chem..

[38] Ceccarelli A., Farneti B., Khomenko I., Cellini A., Donati I., Aprea E., Biasioli F., Spinelli F. (2020). Nectarine volatilome response to fresh-cutting and storage. Postharvest Biol. Technol..

[39] Liu Q., Sun K., Zhao N., Yang J., Zhang Y., Ma C., Pan L., Tu K. (2019). Information fusion of hyperspectral imaging and electronic nose for evaluation of fungal contamination in strawberries during decay. Postharvest Biol. Technol..

[40] Feng L., Zhang M., Bhandari B., Guo Z. (2018). A novel method using MOS electronic nose and ELM for predicting postharvest quality of cherry tomato fruit treated with high pressure argon. Comput. Electron. Agric..

