# Supplementary material for: Analysis of Nutrients and Volatile Compounds in Cherry Tomatoes Stored at Different Temperatures
Source: Foods. 2022 Dec 20;12(1):6. doi: 10.3390/foods12010006 (PMC9818793; doi:10.3390/foods12010006)
Supplement: Supplementary file 1 [file foods-12-00006-s001.zip › foods-2062564-supplementary.pdf]

Table S1. Summary of volatile compounds in cherry tomato during storage.

Serial Number	RT	Identified Compounds	PubChem CID
1	20.02	trans-2-Heptenal	5283316
2	23.31	(E)-2-Octenal	5283324
3	25.52	trans,trans-2,4-Heptadienal	5283321
4	26.79	Benzaldehyde	240
5	29.49	beta-Cyclocitral	9895
6	30.11	Benzeneacetaldehyde	998
7	31.95	Citral	638011
8	25.32	Decanal	8175
9	21.94	Nonanal	31289
10	32.70	(2E,4E)-deca-2,4-dienal	5283349
11	24.25	2,5-Dihydroxybenzaldehyde	70949
12	34.32	2,4-Dimethylbenzaldehyde	61814
13	38.52	5-Heptyldihydro-2(3H)-furanone	7714
14	30.36	3-Cyclohexene-1-propanal	90847
15	16.25	trans-2-Hexenal	5281168
16	26.41	Linalool	6549
17	30.97	Dihydrocarveol	12072
18	35.24	Benzyl alcohol	244
19	35.99	Phenylethyl Alcohol	6054
20	44.94	1-Heptatriacotanol	537071
21	14.44	2-Cyclohexen-1-ol	13198
22	27.15	1,2,6-Hexanetriol	7823
23	28.42	3,3,5-Trimethyl cyclohexanol	8298
24	31.24	2-Isopropenyl-5-methyl-6-hepten-1-ol	556975
25	21.93	2-Propylcyclohexanol	138614
26	26.67	5-methyl-2-prop-1-en-2-ylcyclohexan-1-ol	24585
27	28.41	2-propan-2-ylcyclohexan-1-ol	95331
28	30.98	alpha-Terpineol	17100
29	21.43	3-Cyclopentyl-1-propanol	69842
30	36.17	(-)-Isolongifolol	16217350
31	28.85	2-Butylcyclohexanol	142051
32	31.77	(2E)-2-methyl-6-methylideneocta-2,7-dien-1-ol	5319723
33	28.93	2,6-dimethylcyclohexan-1-ol	21428
34	31.78	Carveol	7438
35	42.92	2,3-Bornanediol	565281

36	43.48	(2,2,6-Trimethyl-bicyclo[4.1.0]hept-1-yl)-methanol	535115
37	29.19	1-methyl-4-prop-1-en-2-ylcyclohexan-1-ol	8748
38	33.82	cis,trans-5,9-Cyclododecadiene-cis-1,2-diol	44630179
39	36.82	7-(Tetrahydro-2H-pyran-2-yloxy)-2-octyn-1-ol	558995
40	10.91	1-Penten-3-one	15394
41	14.17	2-sec-Butylcyclopentanone	558511
42	20.21	6-Methyl-5-hepten-2-one	9862
43	34.49	Nerylacetone	1713001
44	36.59	Irisone	5282108
45	37.69	1-(2,6,6-Trimethyl-2-cyclohexen-1-yl)acetone	579163
46	28.85	3,5,5-trimethylcyclohex-2-en-1-one	6544
47	34.99	3,4,4-trimethylcyclopent-2-en-1-one	520476
48	9.74	Pentan-3-one	7288
49	17.69	3-Octanone	246728
50	30.34	4-chloro-1-phenylbutan-1-one	253533
51	37.68	3-Buten-2-one,4-(2,2,6-trimethyl-7-oxabicyclo[4.1.0]hept-1-yl)-	90899
52	32.23	1,8-dimethyl-4-propan-2-ylspiro[4.5]dec-7-en-9-one	573024
53	34.82	2-cyclopentylidenecyclopentan-1-one	69995
54	34.55	1,2,3,4,5-Cyclopentanepentol	552295
55	17.25	1-Hepten-3-one	520420
56	27.17	2-Methylallyl 2-methylbutyrate	3019320
57	32.82	methyl octadeca-2,5-diynoate	42151
58	33.47	Methyl salicylate	4133
59	34.82	10-Heptadecen-8-ynoic acid, methyl ester, (E)-	5367407
60	40.46	Felbamate	3331
61	38.52	5-Pentyloxolan-2-one	7710
62	33.82	4,9-Decadienoic acid, 2-nitro-, ethyl ester	5466561
63	37.26	[1,1'-Bicyclopropyl]-2-octanoic acid, 2'-hexyl-, methyl ester	552098
64	26.80	2,5-Dioxopyrrolidin-1-yl) benzoate	716426
65	23.72	6-Methylhept-4-en-1-yl 2-methylbutanoate	91700537
66	18.82	Cyclopropanecarboxylic acid, nonyl ester	550169
67	9.09	2-Ethylfuran	18554
68	14.18	3,4-Dihydro-2H-pyran	8080
69	16.97	2-Pentylfuran	19602
70	31.22	3,6-Dimethyl-2-pyridinamine	578956
71	36.86	2H-Pyran, 2-(7-dodecynyloxy)tetrahydro-	86051
72	15.97	1-Heptoxyheptane	12392
73	11.56	Spiro[2,4]hepta-4,6-diene	136590

74	22.77	Perillen	68316
75	23.64	2-Methylprop-1-enylbenzene	13030
76	16.26	(E)-2,2-dimethyloct-3-ene	5366032
77		Styrene	7501
78	29.62	cis-Thujopsene	6432451
79	21.45	Cis-bicyclo[4.2.0]octane	643590
80	18.82	(1-aminocyclopentyl)methanol	66307
81	17.23	2-Ethyl-4,5-dimethyloxaborolane	550808
82	20.91	2-Ethyl-1,6-dioxaspiro[4.4]nonane	581383
83	20.02	Pentylcyclopentane	19540
84	33.38	3-(4-Methylbenzoyl)propionic acid	244162
85	35.03	2-Methoxyphenol	460
86	43.64	2,5-ditert-butylphenol	79983
87	30.61	cis-Verbenol	164888
88	34.56	D-Mannose	18950
89	27.14	Valeric anhydride	74959
90	24.59	Octadecanoic acid, ethenyl ester	66077
91	18.38	1-Methyl-2-propan-2-ylbenzene	10703
92	16.28	(Z)-3-methylundec-4-ene	5364744
93	20.29	5-Methylideneundecane	544747
94	16.55	4-Methylidene-1-propan-2-ylbicyclo[3.1.0]hexane	18818
95	14.57	3-chloro-3-methylhexane	142659
96	17.41	1,1,2-trimethyl-3-(2-methylpropyl)cyclopropane	544064
97	37.26	trans-Traumatic acid	5283028
98	19.37	Cyclooctane,1-methyl-3-propyl-	550139
99	18.38	1-Methyl-3-propan-2-ylbenzene	10812
100	14.58	1,4-Xylene	7809
101	23.2	(3Z)-3-ethyl-2-methylhexa-1,3-diene	5368955
102	23.72	3,5-Dimethylcyclohexene	143321
103	31.33	17-Octadecynoic acid	1449
104	34.54	Hexanoic acid	8892

RT: Retention time.

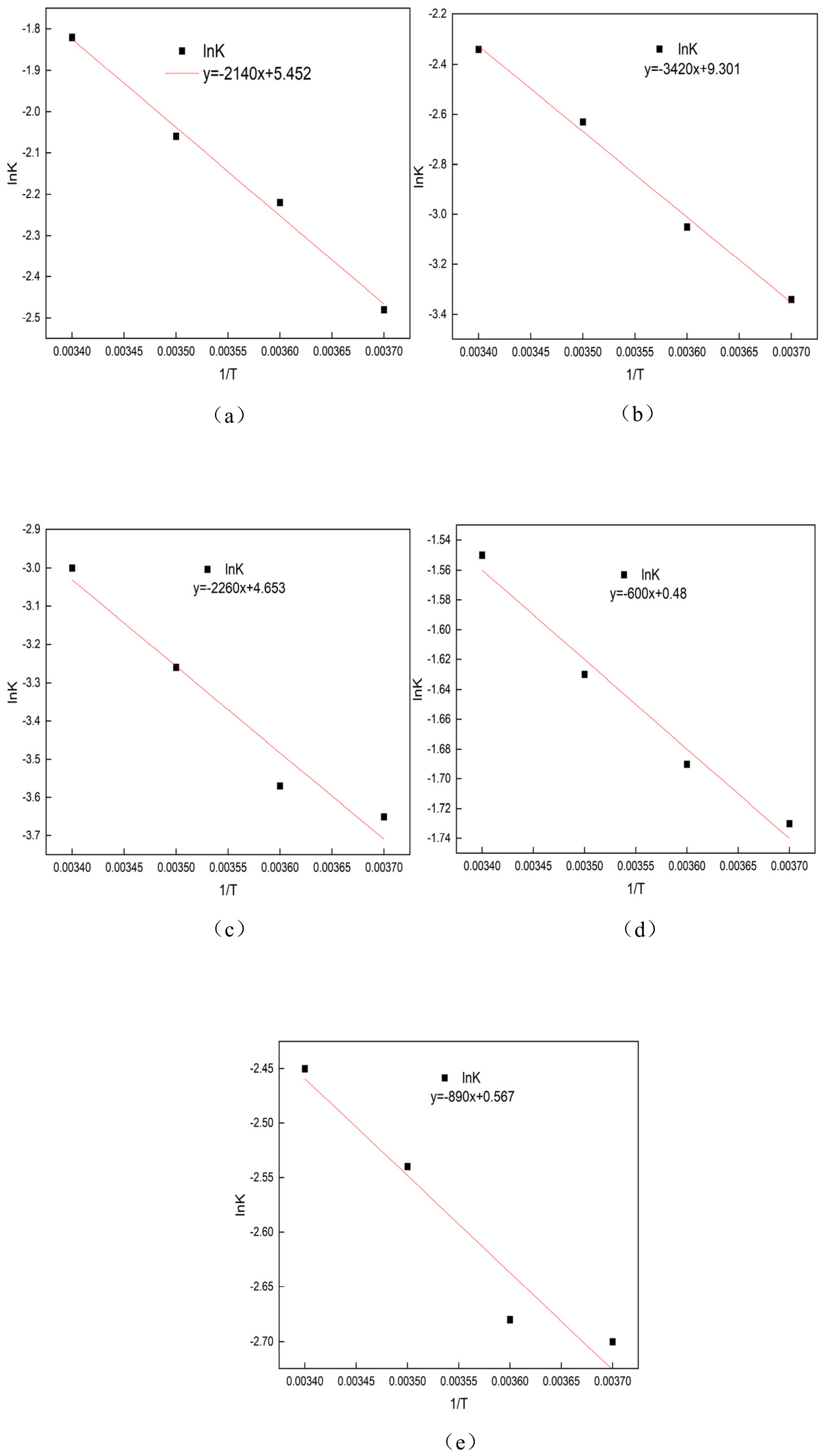

Figure S1. Plot of lnk-1/T of soluble solids (a), reducing sugars (b), titratable acids (c),

ascorbic acid (d) and lycopene (e) of cherry tomatoes at different storage temperatures.

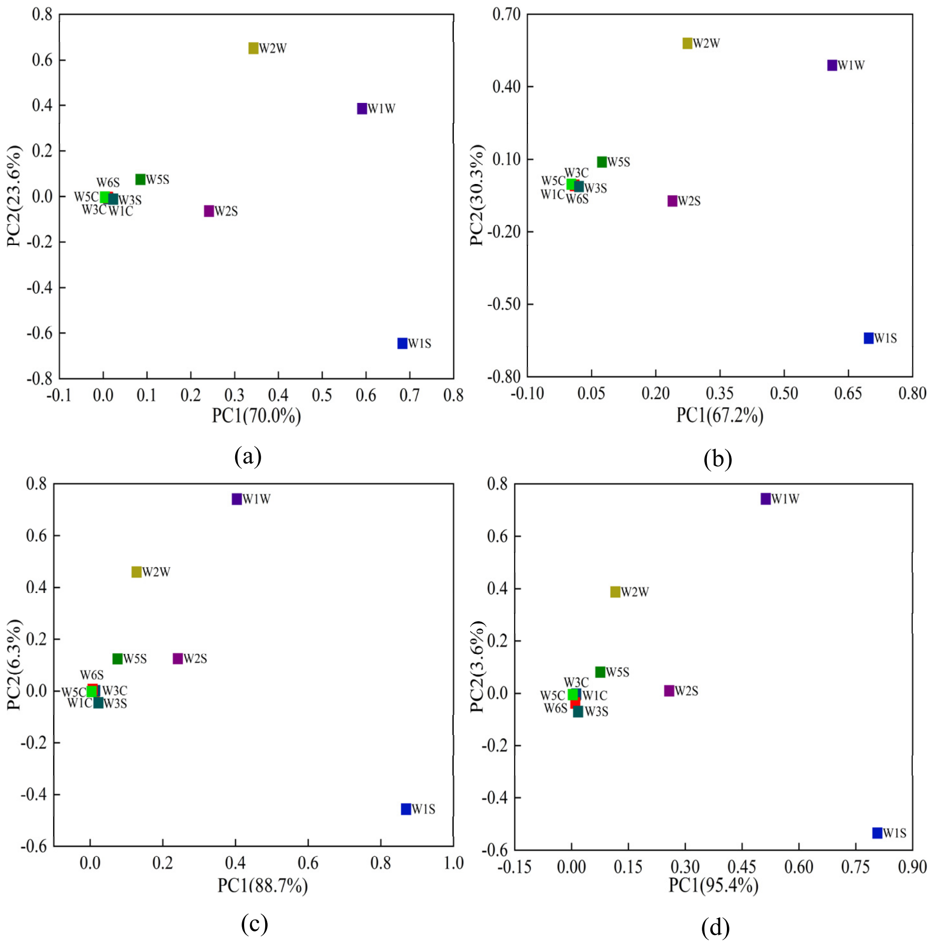

Figure S2. Load diagram of cherry tomatoes stored at 0 (a), 4 (b), 10 (c) or 25 °C (d).
